# Epidemiologic trends for isolated tibia shaft fracture admissions in The Netherlands between 1991 and 2012

**DOI:** 10.1007/s00068-018-01072-3

**Published:** 2019-01-07

**Authors:** Mandala S. Leliveld, Suzanne Polinder, Martien J. M. Panneman, Michael H. J. Verhofstad, Esther M. M. Van Lieshout

**Affiliations:** 1 Trauma Research Unit Department of Surgery, Erasmus MC, University Medical Center Rotterdam, P.O. Box 2040, 3000 CA Rotterdam, The Netherlands; 2 Department of Public Health, Erasmus MC, P.O. Box 2040, 3000 CA Rotterdam, The Netherlands; 3 Consumer Safety Institute, P.O. Box 75169, 1070 AD Amsterdam, The Netherlands

**Keywords:** Tibia shaft fracture, Incidence, Trauma mechanism, Hospital length of stay, Treatment, Years lived with disability

## Abstract

**Introduction:**

Population-based knowledge on the occurrence of specific injuries is essential for the allocation of health care services, optimization of preventive measures, and research purposes. Therefore, the aim of this study was to examine long-term nation-based trends in the incidence rate, trauma mechanism, hospital length of stay (HLOS), treatment, and outcome of hospital-admitted patients with an isolated tibia shaft fracture between 1991 and 2012 in The Netherlands.

**Methods:**

All hospital-admitted patients in The Netherlands between 1991 and 2012 with an isolated tibia shaft fracture were included. Age and gender-standardized incidence rates were calculated for each year. Data were extracted from the National Medical Registration.

**Results:**

The incidence rate for men decreased to 13.8/100,000 person years (py). For women the incidence rate remained stable with 7.2/100,000 py. Incidence showed a peak for adolescent men (15–19 years), and increased in both genders from 65 years onwards. Since 1993 the mean HLOS for isolated tibia fractures reduced from 10.8 to 5.4 days. Mean HLOS increased with age. Mean years lived with disability (YLD) was 4.5 years, declined linearly with age, and showed no gender effect.

**Conclusions:**

In 22 years, the incidence rate of hospital admitted patients with an isolated tibia shaft fracture in The Netherlands dropped with 12%, which was mainly attributable to a 15% decline among men. Incidence rate, trauma mechanism, and HLOS were age and gender related. HLOS also reduced over time. Operation rate and YLD were only age related.

## Introduction

Trauma is among the leading causes of (temporary) disability, dependence, and absence from work. Whereas the incidence rate of long bone fractures in the upper extremities (including humeral fractures) have increased over time [1], the opposite seems the case for long bone fractures in the lower extremities. Literature data for tibia shaft fractures suggest that the incidence rate has declined over the past decades. Weiss et al. performed a nationwide study in Sweden and showed a decrease from 18.7/100,000 person years in 1998 to 16.1/100,000 in 2004 [2]. Prospectively gathered data by Clement et al. from the Royal Infirmary of Edinburgh (United Kingdom) showed the incidence rate in the elderly (65 years or older) declined from 27.0/100,000 in 1990 to 14.0/100,000 in 2004 [3].

Tibia shaft fractures have a bimodal distribution with a peak in young males and in older females [2, 4, 5]. Elderly often sustain such a fracture after a simple fall, in younger patients they are mostly caused by a sports accident. High-energy tibia fractures are usually the result of traffic accidents, such as motor vehicle, pedestrian versus car bumper or cycling collisions [2, 4].

Whether or not the decline in tibia shaft fractures incidence rates and the age and gender relatedness also apply to The Netherlands has not been published. Population-based knowledge on the occurrence of specific injuries is essential for the allocation of health care services, optimization of preventive measures, and research purposes; it may also provide a forecast for the future. Therefore, the aim of this study was to examine long-term population-based trends in the incidence rate, trauma mechanism, hospital length of stay, treatment, and outcome of patients with a tibia shaft fracture (with or without fibula fracture) admitted to a hospital in The Netherlands between 1991 and 2012.

## Methods

### Data source

For this retrospective, epidemiological study data were collected for patients admitted to a hospital in The Netherlands with a tibia shaft fracture with or without a concomitant fibula fracture in the period 1991–2012. The methods are essentially the same as published before [1, 6, 7]. In 2012 The Netherlands had 16.7 million inhabitants [8]. Injury cases were extracted from the National Medical Registration (LMR) of the Dutch Hospital Database (DHD), Utrecht, The Netherlands. The DHD collects hospital data of all hospitals in The Netherlands with a uniform classification system and has an almost complete national coverage (missing values < 5%, except in 2007 12%). These figures were extrapolated by the Consumer and Safety Institute to full national coverage for each year. The extrapolation factor was estimated by comparing the adherence population of the participating hospitals with the total Dutch population in each year using the population data obtained from Statistics Netherlands [9]. Patients are included in the LMR for their main diagnosis at hospital discharge, defined by the International Classification of Diseases (ICD) 9th and (since 2010) 10th revision [10]. Codes for tibia shaft fractures are presented in Table 1. Both patients with and without concomitant fibula (shaft) fracture were included in this study.

**Table 1:** Tibia shaft fractures classified in ICD-9 and ICD-10

ICD	Group	Subgroup	Description
ICD-9	823.2		Fracture of shaft of tibia and fibula closed
		823.20	Closed fracture of shaft of tibia alone
		823.21	Closed fracture of shaft of fibula alone
		823.22	Closed fracture of shaft of fibula with tibia
	823.3		Fracture of shaft of tibia and fibula open
		823.30	Open fracture of shaft of tibia alone
		823.31	Open fracture of shaft of fibula alone
		823.32	Open fracture of shaft of fibula with tibia
ICD-10	S82.2		Fracture of shaft of tibia
		S82.20	Unspecified fracture of shaft of tibia
		S82.22	Transverse fracture of shaft of tibia
		S82.23	Oblique fracture of shaft of tibia
		S82.24	Spiral fracture of shaft of tibia
		S82.25	Comminuted fracture of shaft of tibia
		S82.26	Segmental fracture of shaft of tibia
		S82.29	Other fractures of shaft of tibia

*ICD* International Statistical Classification of Diseases and Related Health Problems

The study was exempted by the local Medical Research Ethics Committee Erasmus MC (no. MEC-2015-218).

### Calculation of incidence rates

Age- and gender-specific incidence rates were calculated in 5-year age groups for each year of the study. In order to adjust for differences in the demographic composition over time, incidence rates were standardized for age (in 5-year age groups) and gender using a direct standardization method, as previously described [1]. In short, the age- and sex-specific incidence rates per 100,000 person years were calculated based upon the Dutch mid-year standard population.

### Trauma mechanism, surgical intervention and hospital length of stay

Data regarding trauma mechanism, operation rate and hospital length of stay (HLOS), were extracted from the LMR database for 10-year age categories and for males and females separately. Percentage of trauma mechanisms and mean HLOS were averaged over 5-year intervals from 1993 to 2012 to assess trends over time. For operation rates, data were averaged over a 5-year interval 2007–2012, as earlier data were not available. For HLOS, the total HLOS was calculated by multiplying the mean HLOS per patient with the total number in each age category.

### Years lived with disability

YLD was obtained by linking the incidence data (subdivided into injury diagnosis groupings) with disability information, that is the proportion of injury cases with lifelong consequences, and injury-specific disability weights of temporary and lifelong consequences. The number of years lived with disability (YLD) was calculated from a patient follow-up survey on health care use and functional outcome [11, 12]. The disability weights were derived from empirical follow-up data on the health-related quality of life of individual trauma patients [11], and adjusted for population norms, age and gender [13]. A random sample of patients was invited to complete a survey at 2.5, 5, 9, and 24 months after injury. YLDs were calculated in three steps [13]. First, data were gathered on the incidence rate, age, and gender distribution of patients hospitalized due to a tibia shaft fracture. Second, the incidence data were divided into the injury categories of the EUROCOST classification system [14]. Finally, the grouped incidence data were combined with the disability weights and durations developed within the framework of the European INTEGRIS (Integration of European Injury Statistics) study [13]. The disability weight reflects the impact of a health condition in terms of health-related quality of life; it has a value ranging from 1, indicating worst imaginable health state, to 0, indicating full health [13].

Registered cases were multiplied with the 1-year disability weight, the proportion of lifelong consequences, and the duration (life expectancy at age of injury, by gender). The mean 1-year disability weights included the temporary and lifelong consequences for cases seen in EDs and those recorded in hospital discharge registers. Data gathered during the 5-year interval 2007–2012 were used for this study.

## Results

### Incidence rates

**Fig. 1:**
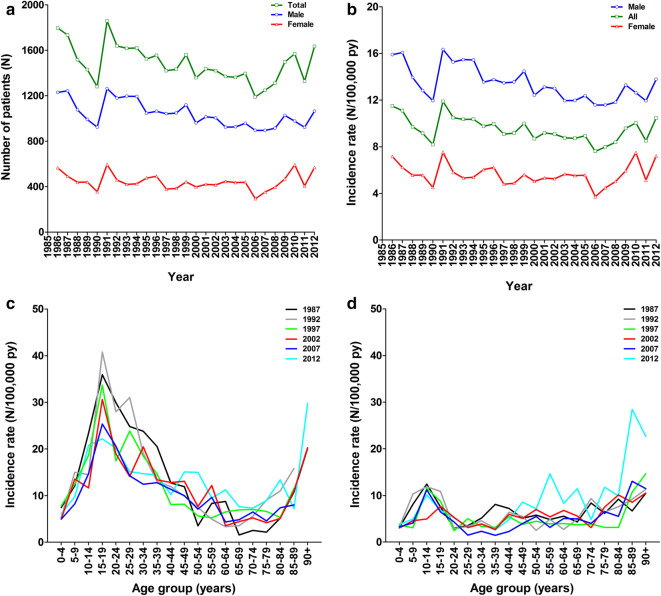
Case numbers (**a**), incidence rates (per 100,000 person years) of isolated tibia shaft fractures annually since 1991 (**b**), and age relation in males (**c**) and females (**d**). **a, b** The crude numbers of patients and the incidence rate per year from 1991 to 2012, respectively. The age-dependency of the incidence rate for every fifth year during the study period in males (**c**) and females (**d**)

Between 1991 and 2012, 32,350 patients required admission for an isolated tibia shaft fracture in The Netherlands. During this period the overall crude number of patients (male and female combined) per year decreased with 12%; from 1860 in 1991 to 1640 in 2012. This decline in crude numbers was most apparent between 1991 and 2006 (− 36%). From 2007 onwards numbers seem to rise again (Fig. 1a).

Figure 1b shows the annual incidence rate of isolated tibia shaft fractures per 100,000 person years (py). For men, the mean incidence rate between 1991 and 2012 was 13.3/100,000 py. The incidence rate decreased with 15% (from 16.3/100,000 py in 1991 to 13.8/100,000 py in 2012). The incidence rate for women remained constant over time: 7.5 per 100,000 py in 1991 and 7.2 per 100,000 in 2012 (mean incidence rate 5.6/100,000 py). The male:female ratio remained 2.4:1 during the study period (Fig. 1a, b).

The age-specific incidence rates showed a bimodal distribution as can be seen in Fig. 1c, d. The incidence shows a first peak among adolescent men (15–19 years). This peak has decreased with 53% during the study period (from 47.5/100,000 py in 1991 to 22.2/100,000 py in 2012). In women, the first peak in incidence is lower than in men. Moreover, it occurred at a younger age (10–14 years) and decreased less than in men (− 38%; from 16.2/100,000 py in 1991 to 10.1/100,000 py in 2012). The gradual increase in incidence rates as seen from 65 years onwards seems unrelated to gender.

Table 2 shows that the incidence rates in the overall population in 2012 was hardly related to age. However, incidence rate showed a clear decrease with age in males (from 15.3 to 8.9/100,000 py) and a noticeable increase with age in females (from 6.5 to 11.7/100,000 py).

**Table 2:** Incidence rates (per 100,000 person years) of isolated tibia shaft fractures in 2012 for different age groups in males and females

Age group	Males	Females	Total (males + females)
0–19 years	15.3	6.5	11.0
20–49 years	15.0	4.7	9.9
50–64 years	12.0	10.0	11.0
≥ 65 years	8.9	11.7	10.5
All ages combined	13.8	7.2	10.5

### Trauma mechanism

**Fig. 2:**
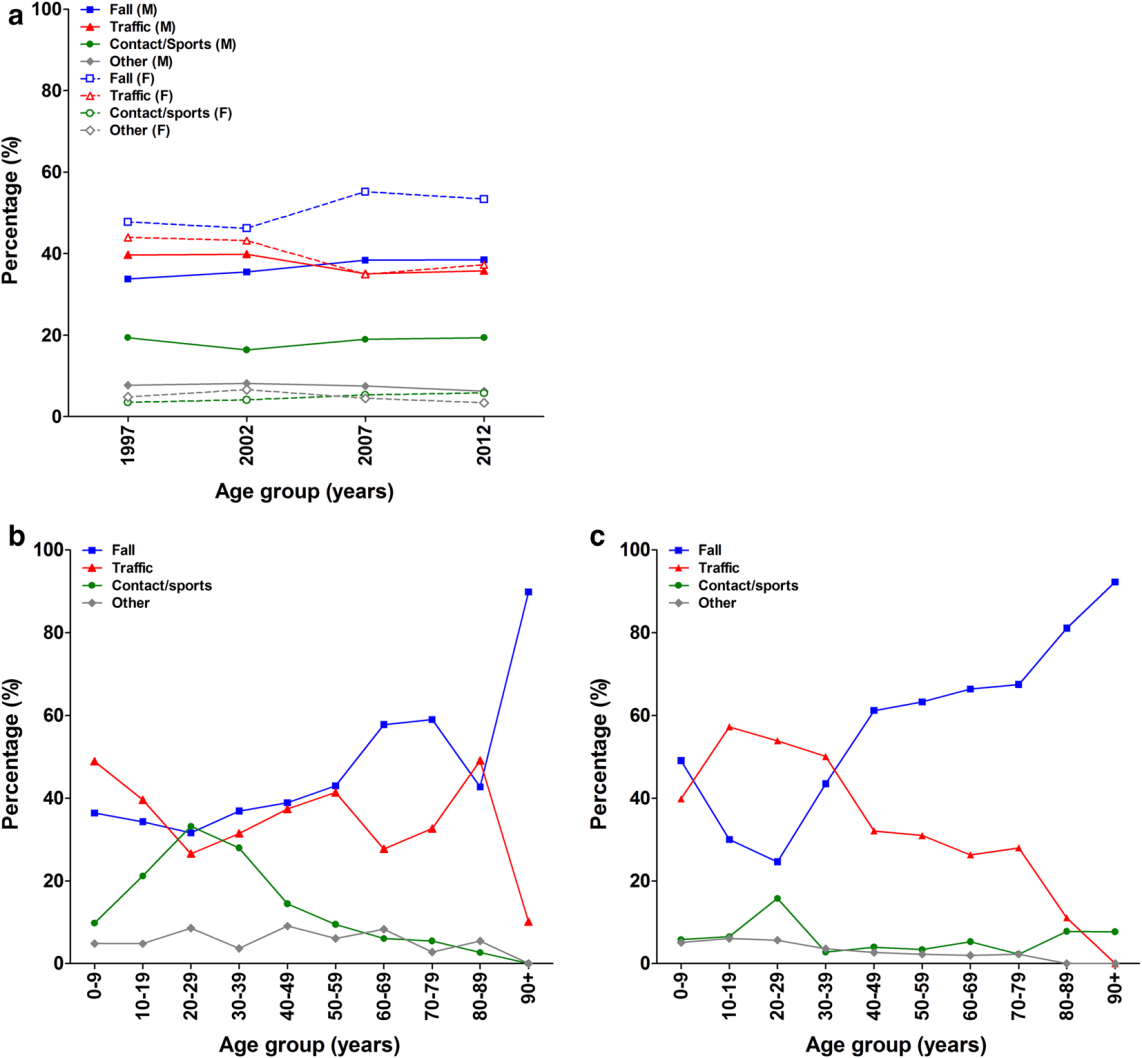
Trauma mechanisms causing isolated tibia shaft fractures. **a** The relative occurrence of trauma mechanisms in four time periods in males and females. The age-dependency of trauma mechanisms in 2012 for males (**b**) and females (**c**)

Figure 2a shows the trauma mechanisms causing isolated tibia shaft fractures in men and women in four consecutive 5-year periods. Throughout the years the majority of fractures in women were caused by a fall. In men traffic accidents and falls contributed almost equally. Sport accidents were seen more in men. During the displayed years, all mechanisms showed fairly steady patterns. The age and gender dependency of trauma mechanisms for 2012 are presented in Fig. 2b, c. In males, the dominant trauma mechanism was a traffic accident until 20 years of age (36%). From 60 years onward, falls predominated (Fig. 2b). Women showed a similar pattern; the dominant trauma mechanism was a traffic accident in the age group < 40 years (50%) and falls in the age group > 40 years (72%; Fig. 2c). Direct contact and sports particularly contributed at the age of 20–29 years, especially in men (27%).

### Operative treatment

**Fig. 3:**
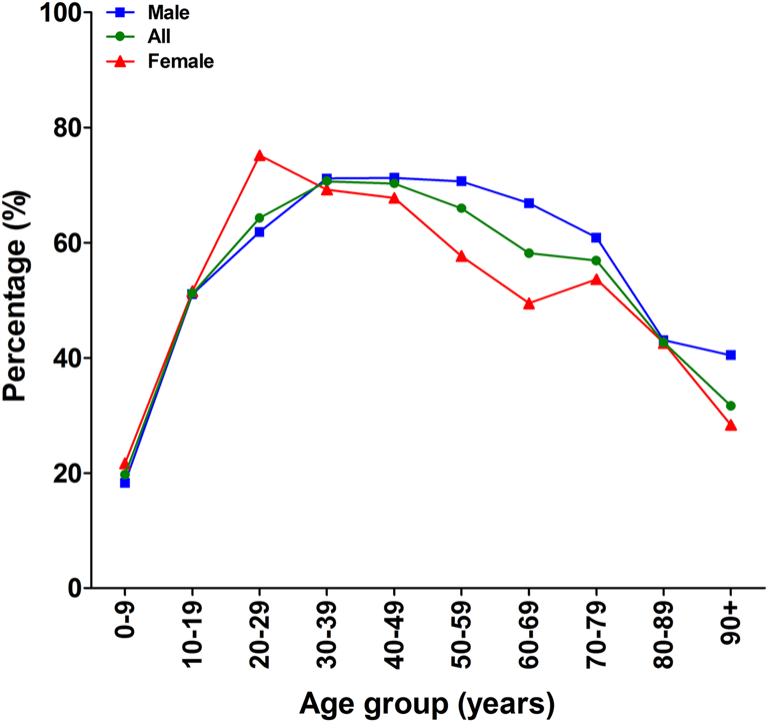
Age-related percentage of patients undergoing surgical treatment in males and females in 2012

In 2012, 57.9% of patients (all ages combined) were treated surgically for their sustained isolated tibia shaft fracture (Table 3). The highest operation rate was 70.7% for patients between the age of 30 and 39 years (Fig. 3). The lowest rates were seen in children (19.7% between 0 and 9 years) and elderly (31.7% in 90+). Operation rate seemed unaffected by gender (Fig. 3).

**Table 3:** Age-related percentage of patients undergoing surgical treatment in males and females in 2012

Age group	Males	Females	Total (males + females)
0–19 years	43.1	40.8	42.4
20–39 years	66.0	72.1	67.3
40–59 years	71.0	62.4	68.2
≥ 60 years	61.0	47.2	53.1
All ages combined	59.9	53.8	57.9

### Hospital length of stay

**Fig. 4:**
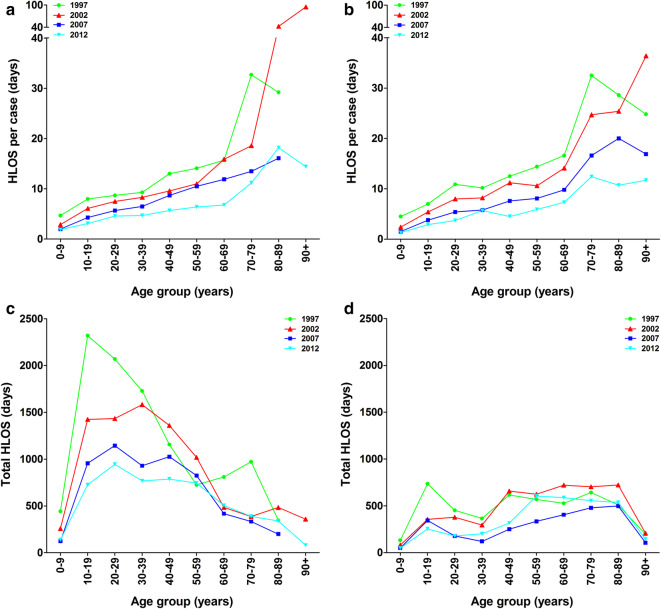
Hospital length of stay due to an isolated tibia shaft fracture. HLOS per patient for four different time periods in males (**a**) and females (**b**). The total HLOS for males (**c**) and females (**d**) for four different time periods

Hospital length of stay (HLOS) per case increased with age. Mean HLOS was 1.6 (SD 4.0) days for patients aged 0–9 years and 12.7 (SD 12.4) days for patients aged 80–89 years in 2012. The mean HLOS per case declined from 10.8 (SD 13.2) days in 1997 to 5.4 (SD 8.6) days in 2012 (Fig. 4a, b). HLOS per case seemed unrelated to gender (Table 4).

**Table 4:** Age-related hospital length of stay due to an isolated tibia shaft fracture in males and females in 2012

Age group	Males	Females	Total (males + females)
0–19 years	2.8	2.4	2.7
20–39 years	4.6	4.6	4.6
40–59 years	6.0	5.3	5.7
≥ 60 years	9.9	9.7	9.8
All ages combined	5.1	6.0	5.4

Due to a higher incidence rate of isolated tibia shaft fractures, the total HLOS was higher in men than in women until 60 years of age. A decline in total HLOS throughout the years was seen in men aged ≤ 60 years (Fig. 4c). The greatest decrease was seen in men aged 10–19 years (− 69%, from 2320 days in 1997 to 725 days in 2012). In women total HLOS was similar for all ages over the consecutive years (Fig. 4d).

### Years lived with disability

**Fig. 5:**
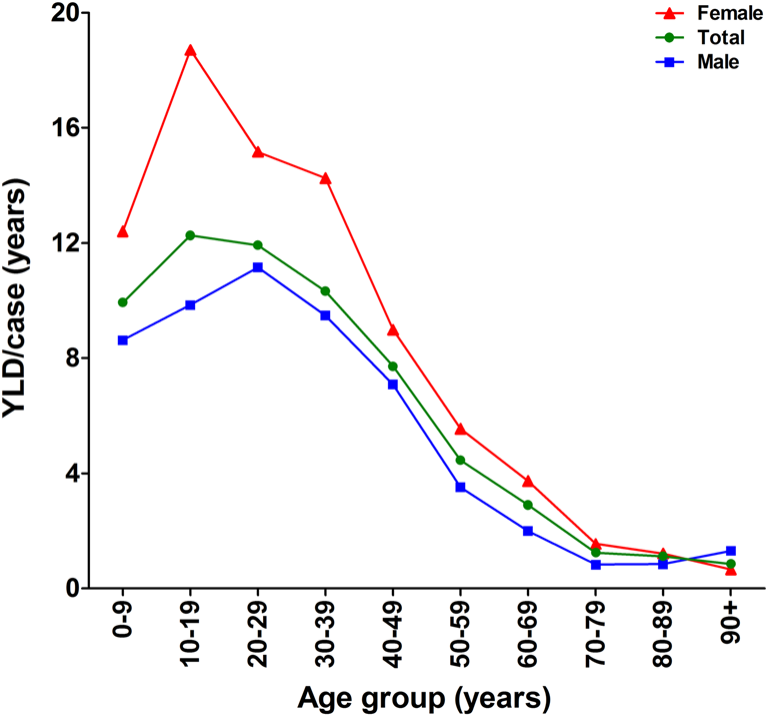
Age-related effect of isolated tibia shaft fractures on the years lived with disability in 2012

Years lived with disability (YLD) per case declined almost linearly with age, but seemed unrelated to gender (Fig. 5). In 2012, the mean YLD per case declined from 7.8 YLD in patients aged 0–10 years to 0.6 YLD in the very elderly (90+ years). The overall YLD for all age groups and both genders combined was 4.5 YLD.

## Discussion

From 1991 to 2012 the incidence rate of hospital admitted patients with isolated tibia shaft fractures dropped with 12% in The Netherlands. This study reports on age- and gender-specific trends in incidence rate, trauma mechanism, hospital length of stay and treatment for patients admitted with these fractures in The Netherlands between 1991 and 2012. Furthermore, this study is the first to describe the age- and gender-specific outcome (YLD) after a sustained tibia shaft fracture.

With 13.3/100,000 py for men and 5.6/100,000 py for women, the overall incidence rate of hospital admissions for isolated tibia shaft fractures in The Netherlands in both genders is lower than the incidence rate of hospital admissions in Sweden reported by Weiss et al. (21/100,0000 py in men and 13/100,000 py in women) [2]. In the National Medical Registration database only the most serious injury of the patient admitted to the hospital is registered. The numbers reported in the current paper therefore represent the actual number of patients admitted for an isolated tibia shaft fracture. The overall decline in incidence rate between 1991 and 2012 in the current study was mainly attributable to a 15% fall in incidence rate in men (to 13.8/100,000 py in 2012). Especially in adolescent men (15–19 years) the incidence rate dropped substantially with 53% during this period. A reduction of 12% in the number of hospital admissions between 1998 and 2004 is also described for the Swedish population [2], but age-dependent incidence trends are not reported by Weiss et al.

The mean hospital length of stay per case decreased by half between 1993 and 2012 both in men and women, whereas the total admission duration decreased only in men. The latter reflecting the fall in incidence rate for men only. Only the most serious injury on admission is listed in The National Medical Registration database. When, for example, a patient is admitted with traumatic brain injury and a tibia shaft fracture, only the brain injury will be registered. Since at least 32.7% of patients with a tibia fracture is multiply injured [15], these injuries will consequently have influenced reporting on the hospital length of stay in other studies [2]. The hospital length of stay in our study thus represents admission days for an isolated tibia shaft fracture only.

The current study is the first to report age- and gender-specific outcome of YLD after isolated tibia shaft fractures. With 26.6%, nature-of-injury categories fracture of patella, tibia, fibula or ankle contributed most to the global YLDs of injury in 2013 [16]. With regard to serious road injuries in The Netherlands during 2000–2009, the total burden of injury was highest for fractures in knee and lower legs (22%) [17]. These findings emphasize that although the incidence of lower leg fractures is low compared with, e.g., hip and wrist fractures, the (lifelong) impact of this injury is high. The high average burden is due to the high disability weights and proportions of lifelong disabilities of some lower leg injuries in combination with the casualties’ relatively young age.

Treatment of individual fractures is not specifically improved by epidemiological studies, but surgeons should have knowledge of the spectrum of fractures which they treat; not only for educational purposes, but also to allow resources to be allocated. A limitation of this study is that data from the National Medical Registration did not provide a subdivision of open and closed tibia fractures. Since the start of the registration, the treatment concept of tibia shaft fractures has changed radically, especially in the last two decades. As reflected in several international guidelines, the fracture itself is more and more seen as a part of a lower leg injury. The prognosis and therapeutic choices are merely depending on the concomitant soft tissue injury. In addition, the provided data concerning the treatment of tibia fractures were only divided in operative versus nonoperative treatment. It does not provide information on trends in the use of different surgical devices (i.e., intramedullary nails, plates or external fixators). Allocation of parameters on the severity of soft tissue injury and implants used are helpful to predict future requirements for equipment and resources such as plastic reconstructive surgery and rehabilitation medicine. A detailed registration of both diagnosis and treatment at the beginning, combined with technical solutions to guarantee the privacy of individual patients, would make a detailed nationwide survey feasible. The Netherlands is currently working on such improvements.

Data registered by The National Medical Registration has an almost complete national coverage and was extrapolated to full national coverage, making it a reliable source for an epidemiological study. However, the database has some limitations. Nonoperatively treated patients, who were not admitted, are not taken into account. This implies that both patients treated in an outpatient clinic only and patients sustaining at least one more severe injury than a tibia shaft fracture are missing. Furthermore, patients are included in the LMR for their main diagnosis at hospital discharge. If the tibia fracture coincides with a more severe trauma or the postoperative course is complicated by, for example, a pulmonary embolism, this additional, more severe diagnose will be registered in the LMR at discharge. This accounts for an underestimation of isolated tibia fractures of less than 10%. Finally, bilateral tibia shaft fractures are included in the database, but not separately listed. If and how these shortcomings influence ratios between men and women and/or the young and elderly, cannot be appraised. On the other hand, since the registration rules have been consistent over time, it is likely that trends during the years are reliable.

## Conclusion

From 1991 to 2012 a total of at least 32,350 patients were admitted for a tibia shaft fracture with or without concomitant fibula shaft fracture to a hospital in The Netherlands. During this period the incidence rate of hospital admitted patients dropped with 12%, which was mainly attributable to a 15% decline in incidence rate in men. Throughout the years the majority of tibia shaft fractures in women were caused by a fall. In men traffic accidents and falls contributed almost equally. No historical trends were visible for the different trauma mechanisms. In both men and women hospital length of stay (HLOS) per case increased with age and the mean HLOS per case declined to 5.4 days in 2012. Operation rate was only age related. The mean YLD was 4.5 years, declined linearly with age and showed no gender effect.
